# Transaxillary three-dimensional endoscopic segmental ductal resection for pathological nipple discharge: a case report

**DOI:** 10.3389/fmed.2026.1936119

**Published:** 2026-09-25

**Authors:** Yi Wang, Pan Li, Yangyang Guan, Yu Zhou, Tao Han, Fei Fan

**Affiliations:** Department of Thyroid and Breast Surgery, The Affiliated Hospital of Panzhihua University, Panzhihua, China

**Keywords:** breast diseases, endoscopy, intraductal papilloma, minimally invasive surgical procedures, nipple discharge

## Abstract

**Background:**

Pathological nipple discharge often requires surgery to remove the affected duct. The conventional circumareolar incisions are generally satisfactory but may result in local hypoesthesia or in ischemic necrosis of the nipple-areola complex because of neurovascular disruption. Therefore, alternative, minimally invasive techniques to reduce this structural damage are being investigated.

**Case presentation:**

A 30-year-old woman presented with a history of serous unilateral nipple discharge for six months. Mammary ductoscopy indicated an intraductal space-occupying lesion, and this was then marked with precise intraductal staining with methylene blue. A three-dimensional endoscopic-assisted segmental ductal resection was performed through a small incision in the natural axillary fold, utilizing the posterior chest space. This particular technique aimed to prevent direct surgical trauma to the central areolar region. Histopathology and immunohistochemistry after surgery indicated the possibility of a benign intraductal papilloma. Patient's recovery was uneventful without complications. The clinical assessment indicated good maintenance of the papillary-areolar blood supply, no serious hypoesthesia of the skin, and no visible scarring of the breast surface.

**Conclusion:**

The three-dimensional endoscopic-assisted axillary approach may be a useful and aesthetically favorable alternative for managing benign intraductal lesions. It may decrease local postoperative complications of traditional incisions, but more clinical studies are required to determine its wider application.

## 1 Introduction

Pathological nipple discharge is clinically defined as the intermittent, continuous, or spontaneous discharge of any fluid from the mammary ducts under non-physiological circumstances (1). Such symptoms can be caused by systemic endocrinological disorders or by a side effect of certain drugs, but in a significant number of cases, they are due to intrinsic breast pathologies (2). Intraductal papilloma is often identified as a primary anatomical abnormality among these pathologies (3). The distinction between benign intraductal proliferation and early malignant change on clinical presentation alone can be difficult, and surgical removal of the involved duct is often recommended for both diagnosis and treatment (4).

These intraductal lesions are usually treated with conventional surgery involving incisions through circumareolar and radial approaches (5). These methods are usually effective in achieving proper resective margins, but are associated with some anatomical difficulties (6). Direct incisions in proximity to the central breast may interfere with the fine microvasculature and sensory nerve supply to the nipple-areola complex (7). The main disadvantages of conventional techniques are the occurrence of postoperative morbidities, from localized sensory impairments (numbness, hypoesthesia) to severe cases of areolar ischemic necrosis (8). These aesthetic and functional deficits can have a great impact on the patient's satisfaction and overall well-being (9), and the surgical community is continually seeking new modalities for acceptable lesion removal with minimal collateral tissue damage (10).

Recently, the concept of minimally invasive breast surgery has evolved toward methods that conceal surgical incisions within the axillary crease (11). While previous minimally invasive techniques for intraductal lesions have been explored—such as periareolar endoscopic duct excision and ductoscopy-guided minimal resection—these often still require incisions on the breast envelope, potentially risking local aesthetic compromises (12). Furthermore, although the transaxillary endoscopic approach has been successfully utilized for radical mastectomies and the removal of solid benign tumors (e.g., fibroadenomas), its application for treating non-palpable intraductal lesions remains scarce (13). In the present article, a clinical case is presented which was managed using a novel application: three-dimensional endoscopic-assisted transaxillary segmental ductal resection (3D-TA-SDR). By combining preoperative ductoscopy-guided methylene blue mapping with the enhanced stereoscopic magnification of 3D endoscopy, this technique introduces specific modifications for navigating the delicate retro-areolar space. This refined approach is assessed for its potential advantages, including effectively removing the pathological duct, maintaining concealed incisions, and potentially safeguarding the neurovascular networks of the nipple-areola complex via the retro-mammary trajectory.

## 2 Case presentation

A 30-year-old female patient (nulliparous, with no lactation history, and moderate breast volume classified as Cup size B) presented to our department with a six-month history of recurrent unilateral serous discharge from the left nipple that was pale yellow in color. There were no palpable masses or skin abnormalities on physical examination. The extensive breast ultrasound (Figure 1) indicated slightly hypoechoic nodules in the left and right breasts, which were preoperatively coded as Breast Imaging Reporting and Data System (BI-RADS) category 3. These lesions were labeled “category 3” with the above mentioned pathological unilateral discharge, and needed further evaluation to assess the potential for atypical changes.

**Figure 1:**
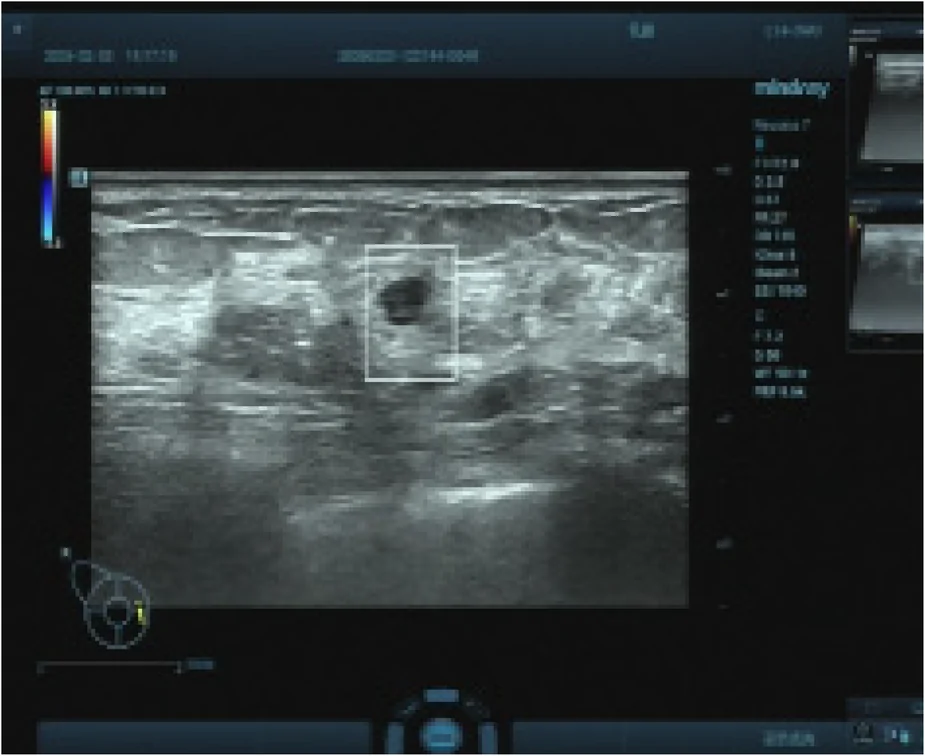
Preoperative breast ultrasound imaging. The sonogram indicates a focal solid breast lesion with low echogenicity. This was combined with the clinical presentation of recurrent nipple discharge and reported as Breast Imaging Reporting and Data System (BI-RADS) category 3, leading to further surgical evaluation.

The direct visualization of the ductal network by mammary ductoscopy indicated a mass in the left breast duct. This ductoscopic identification was immediately followed by the instillation of methylene blue to facilitate precise surgical targeting and the maximization of preservation of adjacent healthy parenchyma. These intraductal growths are usually not palpable and tend to be visually indistinguishable from normal tissue, making intraluminal dye application an important preparatory mapping technique. This visually guided method can guide the removal process, thereby potentially reducing unnecessary traumatic injury to surrounding non-diseased glandular tissue.

An endoscopic segmental resection was planned using a three-dimensional axillary approach, in order to optimize the clinical and aesthetic results for the left breast. Pre-operative surface delineations were mapped (Figure 2), tracking the planned route from the axilla to the retro-areolar area. The patient was placed in a supine position with the left arm abducted to 90 degrees. A 3-cm incision was strategically placed in the natural axillary crease. A working space was established in the retro-mammary layer (between the posterior mammary fascia and the pectoralis major muscle) using blunt and sharp dissection. To maintain the operative cavity, CO2 insufflation was applied at a pressure of 8 mmHg. The 3D endoscopic system (10 mm diameter, 30-degree angled scope) was introduced through a primary 10-mm trocar, accompanied by two 5-mm working trocars for the dissecting instruments. The enhanced depth perception of the 3D system was particularly useful in navigating the small retro-areolar space.

**Figure 2:**
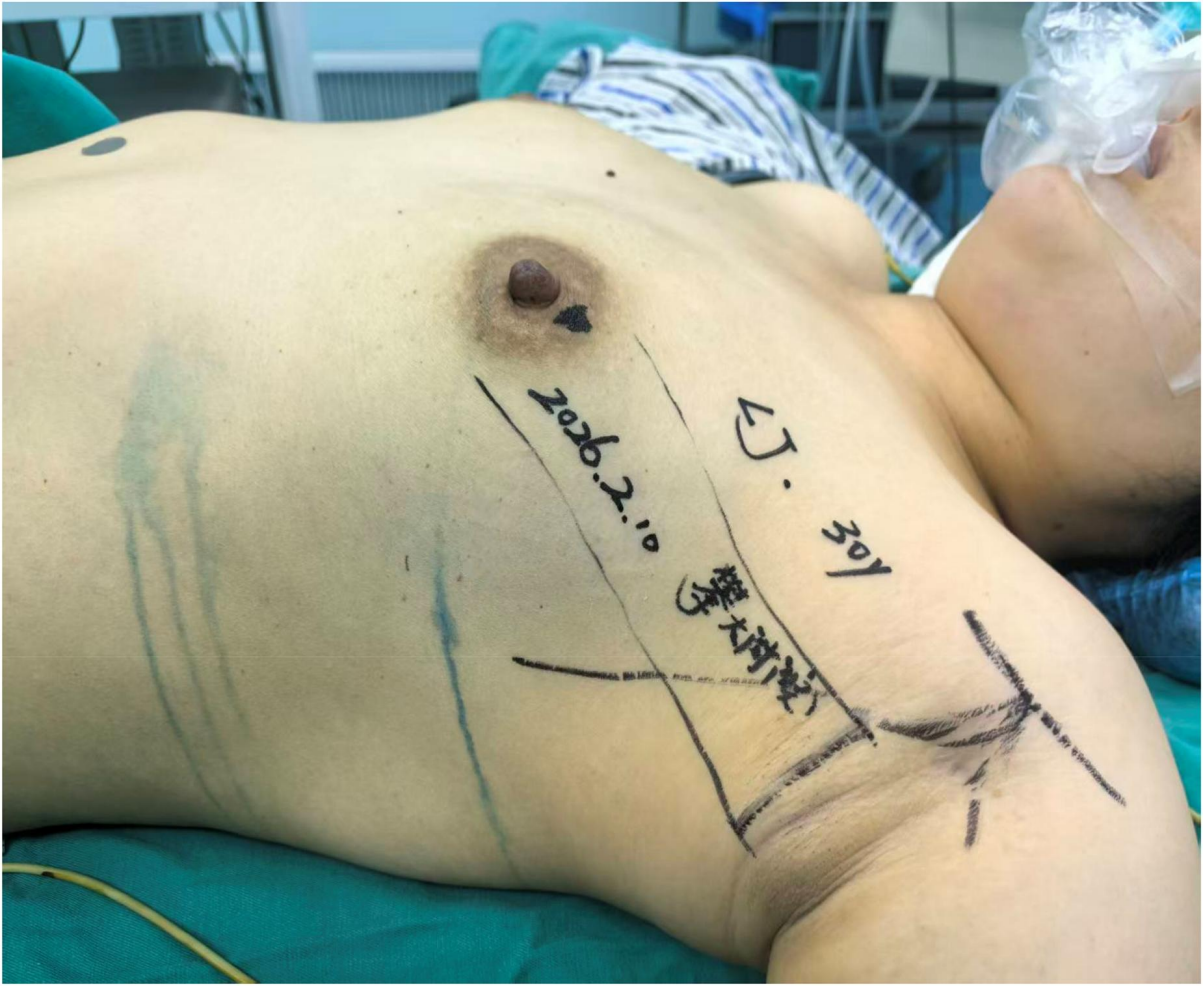
Preoperative body surface markings indicating the planned surgical trajectory. The markings made on the patient's thorax are meant to visually direct the path of the surgical tunnel, which is subcutaneously created from the natural axillary crease to the retro-areolar zone. This surgical plan is established in advance to help ensure that there will be enough room for the endoscopic instruments during the procedure, potentially optimizing the final cosmetic outcome.

The specific, dye-marked duct was carefully mobilized towards the nipple-areola complex (NAC). A critical technical precaution for NAC preservation involved maintaining a safe dissection thickness of at least 5 mm from the nipple skin to protect the subdermal vascular plexus. The surgical team strictly avoided the use of monopolar cautery near the central areolar area, opting for an ultrasonic scalpel and meticulous blunt dissection to minimize thermal spread and excessive traction. The lesion was ligated and excised immediately below the nipple base, and the involved segment was removed with a thin margin of normal glandular tissue, successfully avoiding damage to regional neurovascular networks. The total operative duration was approximately 65 minutes, with an estimated blood loss of 10 mL. The solid mass was completely removed en bloc (Figure 3). Postoperatively, a closed-suction drainage tube was inserted briefly through the axillary incision for the drainage of any possible exudate and for the control of the dead space in the subcutaneous tissue (Figure 4).

**Figure 3:**
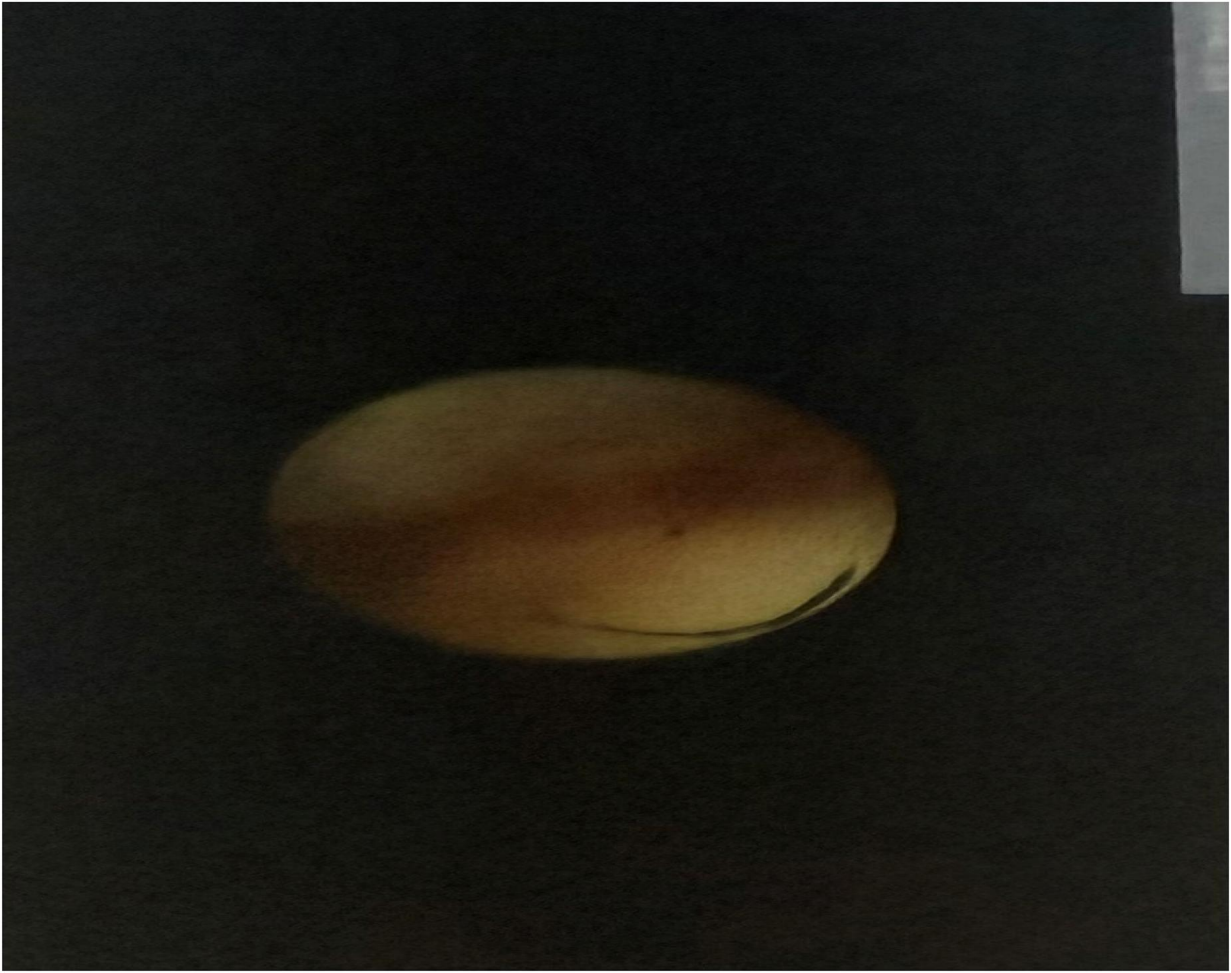
Macroscopic appearance of the excised surgical specimen. The skin incision is strategically located within the natural axillary fold, reducing the possibility of a visible skin scar across the anterior breast envelope. A temporary closed-suction drain is also depicted, designed to evacuate potential postoperative fluid and allow the space between the skin and the underlying tissues to collapse, thereby facilitating optimal healing of the wound and good apposition of the tissues.

**Figure 4:**
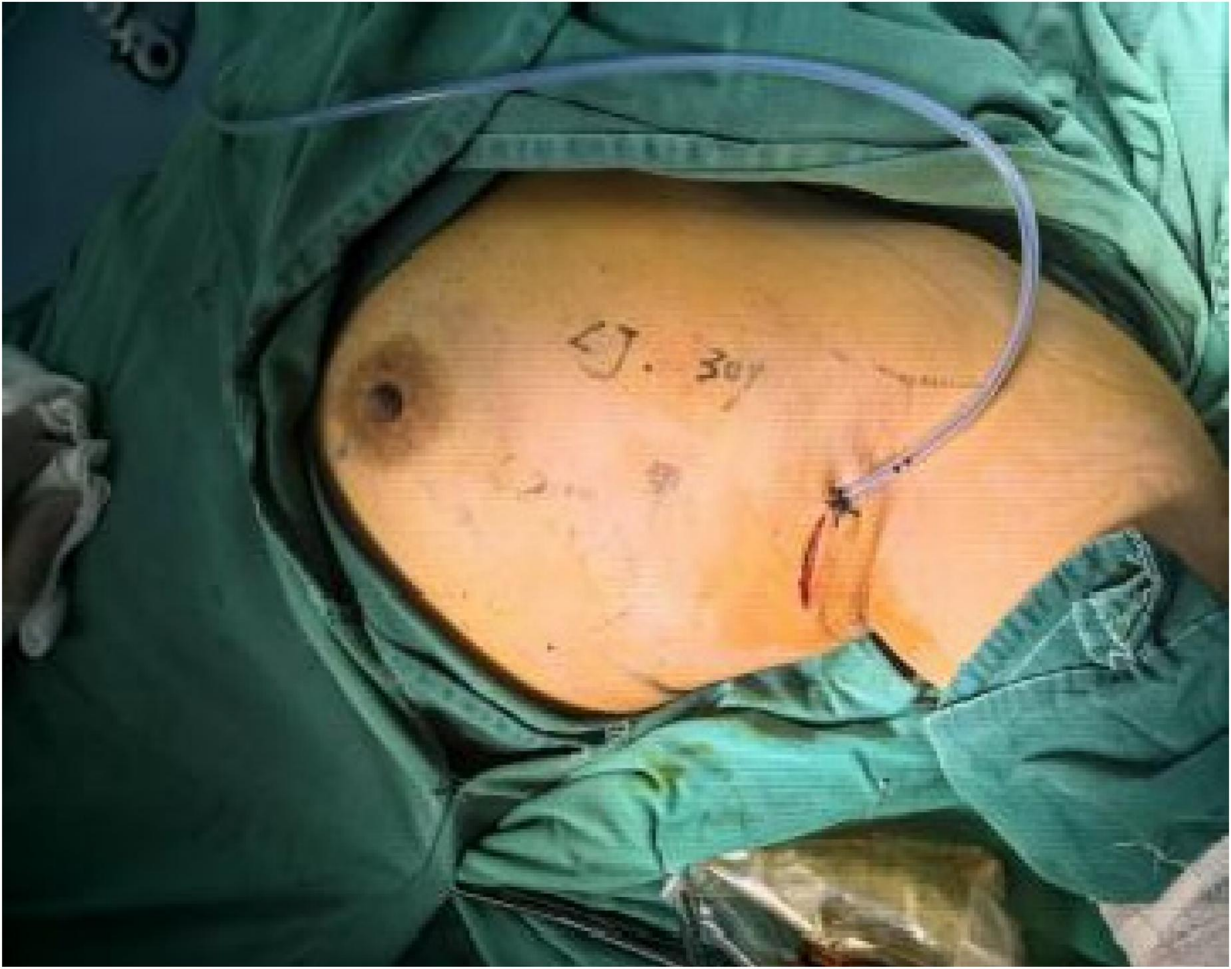
Postoperative view illustrating the axillary incision and the placement of a drainage tube. Proliferation of both the epithelial and structural components is evident in the tissue architecture observed. These particular morphological features are notable when evaluated alongside the immunohistochemical expression pattern, and are consistent with a benign intraductal papilloma.

The postoperative course was unremarkable, and the patient was discharged on postoperative day 2 without complications. Follow-up evaluations were systematically conducted at 1, 3, and 6 months postoperatively. During the 6-month follow-up period, clinical assessments revealed no recurrence of nipple discharge. The patient reported high satisfaction with the aesthetic outcome, citing the complete absence of visible breast scarring, and subjectively reported normal sensory function in the nipple-areola region. Hyperplastic epithelial components were observed in the histopathological examination after surgery (Figure 5). In order to distinguish papillary lesions that may be malignant from a benign papilloma, immunohistochemical staining was performed. The analysis indicated differential positivity of estrogen receptor, progesterone receptor, a score of 1 + for human epidermal growth factor receptor 2, and speckled positivity for cytokeratin 5/6. Most significantly, both P63 and smooth muscle myosin heavy chain were positively expressed, suggesting that the myoepithelial cell layer was still intact. A low Ki67 proliferation index (around 5 percent) was observed. The overall morphological and immunohistochemical features are strongly suggestive of a diagnosis of benign intraductal papilloma.

**Figure 5:**
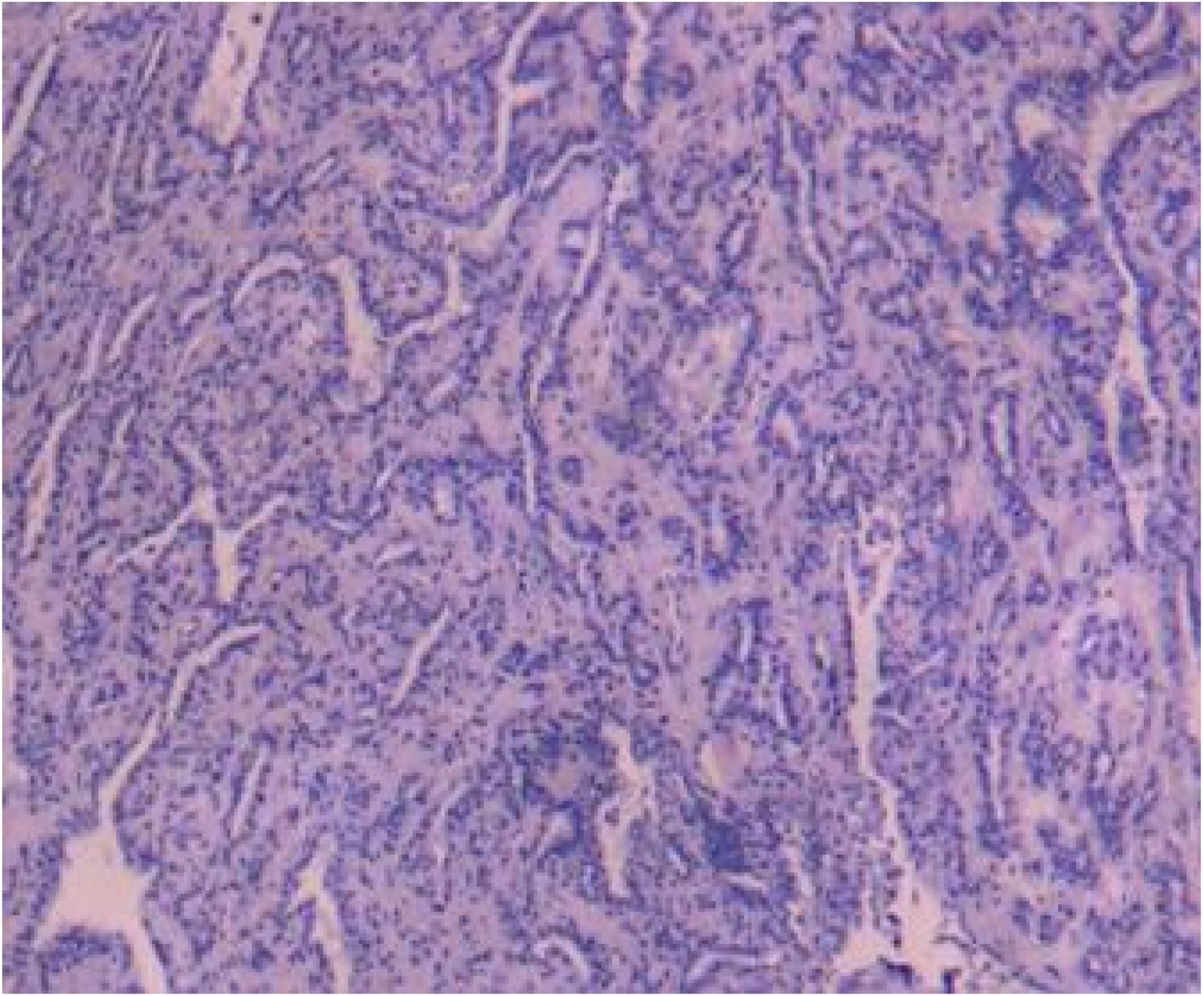
Histopathological evaluation of the resected lesion (hematoxylin and eosin staining, original magnification ×200). The microscopic architecture reveals hyperplastic epithelial and structural components. These morphological findings, in combination with the corresponding immunohistochemical profile, suggest features consistent with a benign intraductal papilloma.

## 3 Discussion

Pathological nipple discharge is a common clinical sign in senology, and is the second most common clinical presentation after breast lumps and mastalgia (14). In some of these presentations, the cause is physiological or systemic, but often intermittent or persistent discharge may be associated with intrinsic abnormalities of the duct(s) (15). Intraductal papilloma is thought to be a major lesion associated with this clinical sign (16). When assessing these clinical characteristics, it is important to keep in mind that they are associated with the underlying atypical pathology and do not themselves cause it (17). However, prompt surgical evaluation is usually recommended because there are some observations to suggest that there is an overlap between persistent discharge and early neoplastic processes (18).

A circumareolar or radial approach is usually employed for conventional surgical management of intraductal lesions (19). These conventional techniques are generally effective for lesion resection, but can have a number of postoperative complications (20). The direct incisions adjacent to the nipple areola complex (NAC) may disturb the local microcirculation and sensory innervation. There is a possibility of hypoesthesia, numbness, and even partial areolar ischemia in severe cases after surgery. All such compromises are cosmetic and functional limitations which may impact overall patient quality of life, and thus remain a subject of study for breast surgery techniques.

Compared with conventional periareolar duct excision, the 3D-TA-SDR technique offers distinct theoretical advantages. While traditional approaches are time-efficient and require minimal specialized equipment, direct periareolar incisions inherently risk local microcirculation disruption and visible scarring on the anterior envelope. Conversely, the 3D-TA-SDR approach utilizes the retro-mammary space, allowing surgical instruments to bypass the superficial structures. Assisted by the unique stereoscopic view and optical magnification of the 3D endoscopic system, this trajectory appears to effectively preserve the critical neurovasculature supplying the NAC. In addition, preoperative ductoscopy and methylene blue localization are essential adjuncts. Because intraductal papillomas may be diminutive and difficult to visually delineate (21), intraluminal dye administration provides a crucial visual guide (22), helping to ensure adequate removal of the affected duct while minimizing collateral damage (23).

However, the preliminary indications and practical limitations of this technique must be explicitly acknowledged. Currently, this approach is best suited for patients with single-duct discharge and sufficient breast volume (e.g., Cup size B or larger) to allow the establishment of an adequate retro-mammary working space. Furthermore, interpreting these promising short-term results requires caution. One notable limitation of our report is the reliance on patient-reported subjective assessments rather than objective sensory tools (such as Semmes-Weinstein monofilaments) to evaluate postoperative NAC function. Additionally, real-world constraints such as a steep surgical learning curve for endoscopic breast procedures, increased operative duration, and the high costs associated with specialized 3D equipment may limit its immediate widespread application. Based on this individual case, the 3D-TA-SDR approach could be a viable alternative for selected patients prioritizing cosmetic outcomes, but larger cohort studies are required to evaluate its broader applicability and long-term efficacy.

## 4 Conclusion

The three-dimensional endoscopic-assisted axillary approach may serve as a potential alternative surgical option for the treatment of pathological nipple discharge from intraductal lesions. This method has the potential to minimize direct surgical trauma to the NAC by using the posterior breast space and preoperative ductoscopy-guided staining. Complete macroscopic lesion resection was achieved in this individual case; however, the long-term oncological adequacy and radicality of this approach remain to be verified in larger cohort studies. As seen in this case, the preservation of local neurovascular structures and the aesthetic outcome appeared excellent and may overcome the potentially negative aspects of the traditional circumareolar approach. However, as this report is a single case, the conclusions drawn from this case should not be interpreted as definitive. Well-designed studies with larger patient cohorts are required to further elucidate the broader applicability, surgical safety profile, and long-term efficacy of this minimally invasive approach. This surgical technique is a preliminary but promising option to achieve optimal cosmetic preservation in selected patients.

## Data Availability

The raw data supporting the conclusions of this article will be made available by the authors, without undue reservation.
